# Proton pump inhibitors: potential cost reductions by applying prescribing guidelines

**DOI:** 10.1186/1472-6963-12-408

**Published:** 2012-11-19

**Authors:** Caitriona Cahir, Tom Fahey, Lesley Tilson, Conor Teljeur, Kathleen Bennett

**Affiliations:** 1 HRB Centre for Primary Care Research, Division of Population Health Science, Royal College of Surgeons in Ireland, 123 St Stephens Green, Dublin 2, Ireland; 2 National Centre for Pharmacoeconomics, St James Hospital, Dublin 8, Ireland; 3 Health Information and Quality Authority (HIQA), George's Court, George's Lane, Dublin 7, Ireland; 4 Department of Pharmacology & Therapeutics, Trinity Centre for Health Sciences, St James Hospital, Dublin 8, Ireland

**Keywords:** Proton pump inhibitors, Cost-effective, Guidelines, Generic

## Abstract

**Background:**

There are concerns that proton pump inhibitors (PPI) are being over prescribed in both primary and secondary care. This study aims to establish potential cost savings in a community drug scheme for a one year period according to published clinical and cost-effective guidelines for PPI prescribing.

**Methods:**

Retrospective population-based cohort study in the Republic of Ireland using the Health Services Executive (HSE) Primary Care Reimbursement Services (PCRS) pharmacy claims database. The HSE-PCRS scheme is means tested and provides free health care including medications to approximately 30% of the Irish population. Prescription items are WHO ATC coded and details of every drug dispensed and claimants’ demographic data are available. Potential cost savings (net ingredient cost) were estimated according to UK NICE clinical guidelines for all HSE-PCRS claimants on PPI therapy for ≥3 consecutive months starting in 2007 with a one year follow up (n=167,747). Five scenarios were evaluated; (i) change to PPI initiation (cheapest brand); and after 3 months (ii) therapeutic switching (cheaper brand/generic equivalent); (iii) dose reduction (maintenance therapy); (iv) therapeutic switching and dose reduction and (v) therapeutic substitution (H2 antagonist).

**Results:**

Total net ingredient cost was €88,153,174 for claimants on PPI therapy during 2007. The estimated costing savings for each of the five scenarios in a one year period were: (i) €36,943,348 (42% reduction); (ii) €29,568,475 (34%); (iii) €21,289,322 (24%); (iv) €40,505,013 (46%); (v) €34,991,569 (40%).

**Conclusion:**

There are opportunities for substantial cost savings in relation to PPI prescribing if implementation of clinical guidelines in terms of generic substitution and step-down therapy is implemented on a national basis.

## Background

Proton pump inhibitors (PPI) are indicated in the treatment of acid related dyspepsia and peptic ulcers and are one of the most frequently prescribed classes of drugs in the world [1]. PPIs ranked as the sixth most frequently dispensed therapeutic class in the US and third in Ireland in 2009 [2,3]. The high volume of PPI prescribing may reflect the superior efficacy of PPIs and the relative lack of adverse drug effects and interactions compared to other acid inhibiting agents. However PPIs cost more than other acid inhibiting agents and the volume of prescribing has had a substantial impact on prescribing budgets worldwide [4,5]. Expenditure on PPIs was €595 million in England in 2006 and €4.5 billion on one PPI (Esomeprazole-Nexium®) in the US in 2009 [6,7]. In Ireland total expenditure on PPIs has increased from approximately €7 million in 1995 to €95 million in 2009. PPIs are one of the most expensive drug groups reimbursed in Ireland accounting for approximately 10% of overall drug expenditure [3].

Prescribing guidelines have been developed in several countries to reduce PPI expenditure and ensure appropriate use. The National Institute of Clinical Excellence (NICE) guidelines on the appropriate use of PPIs in the treatment of dyspepsia recommends regular review of patients to assess the continuing need for PPIs and stepping down to a lower maintenance dose or alternative medication to control symptoms. The guidelines also recommend prescribing the least expensive PPI [8,9]. Studies to date have indicated that guidelines are not being followed with evidence of overprescribing of PPIs in both primary and secondary care. A UK study, found that 24% of patients admitted to hospital were prescribed a PPI in the community and of these only 54% had an appropriate indication for PPI treatment [10]. Studies in secondary care in the US, Australia, New Zealand, Italy, UK and Ireland found 65%, 63%, 40%, 68%, 51% and 33% of hospital inpatients did not have appropriate indication for PPI therapy, respectively [11-16]. In Ireland and Italy, 71% and 66% of PPI prescribing was initiated in hospital [14,16]. In the UK, 54% of PPI prescribing was initiated in hospital and 51% of initiated prescribing was without an appropriate indication [15].

The superior efficacy and safety of PPIs are often used as justification for their use over other acid-suppressing agents but side-effects, though rare, need to be considered. Long term PPI use has been associated with an increase in community and hospital acquired pneumonia, clostridium difficile colitis and fractures [17-21]. Given these associations, limiting PPI use to short term treatment and taking the minimum amount required may be considered prudent in view of clinical uncertainty over long term acid suppression.

Economic modelling has assessed whether the additional cost of PPI therapy compared to other acid inhibiting agents is acceptable given PPIs superior efficacy in healing and relieving symptoms. A cost-effectiveness analysis of long term strategies for managing gastrointestinal symptoms in primary care reported initial treatment with a PPI followed by maintenance therapy with a H2 antagonist to prevent symptomatic recurrence as the optimal strategy [22]. Treatment with H2 antagonists was also the optimal strategy for the prevention of non-steroidal anti-inflammatory drug induced gastro-intestional toxicity [23].

Research to date has focused on the clinical evidence of whether or not the guidelines are being followed and on specific groups of patients in different clinical settings (e.g. hospitals). Economic data is needed to inform health policy makers and practitioners at a national level to guide policy development. Expenditure on PPIs decreased in Northern European countries (England, Scotland and Sweden) between 2001 and 2007, despite an increase in PPI utilisation, through multiple demand side reforms encouraging the prescribing of low cost generic PPIs, such as omeprazole. The exception was Ireland, were both utilisation and expenditure on PPIs increased due to increased prescribing of esomeprazole and decreased prescribing of generic omeprazole [24,25]. If PPI prescriptions were restricted generally to the recommended guidelines, what would the impact be on government drug expenditure? The aims of this study were to: (i) investigate trends in the duration and dose of PPI prescribing in a national community drug scheme in Ireland in a one year period 2007–2008; (ii) determine potential cost savings in a one year period (2007–2008) by examining different scenarios in prescribing patterns of PPIs according to clinical and cost-effectiveness guidelines and (iii) compare potential cost savings stratified by different age groups.

## Methods

### Study population

The National Shared Services Primary Care Reimbursement Service of the Health Service Executive in Ireland (HSE-PCRS) pharmacy claims database of dispensed medications was used to identify the study population. The HSE-PCRS general medical services (GMS) scheme provides free health services including medications to eligible persons in Ireland. The GMS scheme is means tested for those aged less than 70 years and was provided to all those ≥70 years between July 2001 and December 2008. The HSE-PCRS GMS scheme provides free medication to approximately 32% (1,352,120) of the Irish population and covers 74% of state expenditure on medication [3].

The HSE-PCRS pharmacy claims database provides details on monthly dispensed medications for each individual within the scheme. Prescription claims are coded using the World Health Organisation Anatomical Therapeutic Chemical (ATC) classification system [26] and prescriber information, brand name, defined daily doses (DDD), strength, quantity, method and unit of administration of each drug dispensed, ingredient costs and pharmacist dispensing fees per item dispensed are also recorded. Drugs are categorised into four classes: unbranded generic, branded generic, proprietary drug with a generic equivalent and proprietary drug with no generic equivalent. Gender, age group and health board region of each claimant is also recorded, but no diagnosis or outcomes are reported. Ethical approval was not required.

### Estimation of potential cost savings

Five scenarios were identified according to published NICE clinical guidelines for more cost-effective PPI prescribing taking into consideration the appropriate clinical indications for PPI therapy and their relative efficaciousness in the treatment of most acid related gastrointestinal conditions [8,9]. Each scenario estimated potential cost savings by substituting the dispensed PPI for an alternative PPI (e.g. cheaper/generic or lower dose etc.) for all claimants on PPI therapy (ATC code A02BC) for at least 3 or more consecutive months in 2007 with a one year follow up (12 month period for each claimant). The maximum therapeutic dosage and maintenance dosage were evaluated by calculating the prescribed daily dose for each claimant according to details on the DDD, strength, quantity, unit of measurement and pack size of the dispensed PPI for the specified time period. PPI dosage was classified as maximum or maintenance dosage at the end of each month according to the calculated prescribed monthly dose. The maximum therapeutic dosage for PPI prescribing was classified as 40 mg daily for omeprazole, pantoprazole and esomeprazole, 30 mg daily for lansoprazole and 20 mg daily for rabeprazole. Maintenance dosage was classified as 20 mg daily for omeprazole, pantoprazole, esomeprazole, 15 mg daily for lansoprazole and 10 mg daily for rabeprazole.

### Five scenarios for cost-minimisation

1. *Least expensive PPI at initiation* - patients continue on their original dose and quantity for the one year time period

After 3 months of initial therapy

2. *Therapeutic switching* (cheaper brand/generic equivalent) - patients are switched to the least expensive appropriate PPI but they continue on their original dose and quantity for the one year time period

3. *Dose reduction* (maintenance therapy) - patients on PPI therapy at maximum dosage step down to a maintenance dose of their existing PPI

4. *Therapeutic switching and dose reduction* - patients on PPI therapy at maximum dosage step them down to a maintenance dose of the least expensive PPI (double switch)

5. *Therapeutic substitution* - Substitution of patients existing PPI with a H2 Antagonist

Costs were calculated as the net ingredient cost (NIC) of the dispensed PPI and the total expenditure which included NIC and pharmacist dispensing fee. Potential cost savings were determined by comparing the cost of each of the five scenarios to continued PPI use (actual PPI utilisation in the HSE-PCRS pharmacy claims database).The price per dose unit for each PPI was calculated. Potential savings were assessed as total ingredient cost - (units dispensed * substituted PPI price per unit). Claimants were categorised by gender and age groups (16 to >75 years; by 10 year age categories). Data analysis was performed using SAS statistical software package version 9.2 (SAS Institute Inc. Cary, NC, USA) with 95% confidence intervals.

## Results

### Overall trends in PPI prescribing

**Figure 1:**
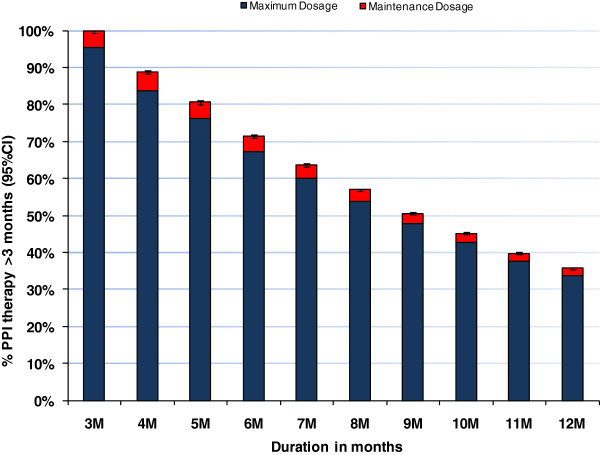
**Duration and dosage of PPI therapy for a one year continuous period for patients on PPI therapy for ≥3 months at maximum therapeutic dosage.** Notes: One year period- January 2007 to January 2008, February 2007 to February 2008. Dosage is the dose at the end of each month. Maximum therapeutic dose= 40 mg/daily omeprazole, pantoprazole and esomeprazole. 30 mg/daily lansoprazole and 20 mg/daily rabeprazole. Maintenance therapeutic dose=10-20 mg/daily omeprazole, 20 mg/daily pantoprazole and esomeprazole. 15 mg/daily lansoprazole and 10 mg/daily rabeprazole.

In 2007 a total of 167,747 patients (13% of the eligible population) were prescribed PPIs for ≥3 consecutive months and 301,961 (24% of the eligible population) were prescribed PPIs intermittently. In this group of patients prescribed PPIs for ≥3 consecutive months, 102,475 (61%) were prescribed PPIs at maximum therapeutic dosage; 3,688 (2%) were co-prescribed two PPIs. Almost three quarters of patients, 73,240 (71%) continued on PPI therapy for 6 consecutive months with 36,555 (36%) on PPI therapy for a one year continuous period. Of those on PPI therapy for a one-year continuous period, the majority 34,589 (95%) continued on maximum therapeutic dose (Figure 1).

### PPI prescribing by age group

Table 1 presents the percentage of patients prescribed PPIs for ≥3 consecutive months in 2007 by age distribution of the HSE-PCRS population and the proportion of those prescribed PPIs at maximum therapeutic dosage. The majority of PPI prescribing for ≥3 consecutive months was in the older age groups (65 years and older) but the proportion of PPI prescribing at maximum dosage was consistent across age groups (approximately 60%).

**Table 1:** Percentage of patients prescribed PPIs ≥ 3 months in 2007 (by age distribution of the HSE-PCRS population)

***Age Bands***	**% ≥ *3 months***	***Proportion at maximum dosage***
16-24 years	1.41	60.46
25-34 years	3.62	63.99
35-44 years	7.32	64.64
45-54 years	14.80	63.68
55-64 years	20.79	62.67
65-69 years	23.87	61.02
70-74 years	23.11	59.12
75+ years	28.87	60.29

**Notes**: Maximum therapeutic dose= 40 mg/daily omeprazole, pantoprazole and esomeprazole. 30 mg/daily lansoprazole and 20 mg/daily rabeprazole.

### Potential cost savings

The total net ingredient cost for patients on PPI therapy ≥3 months in 2007 was €88,153,174; total expenditure (including pharmacist dispensing fee) was €97,391,999. The most frequently prescribed PPI was lansoprazole; a proprietary drug with a generic equivalent at an average monthly cost of €42. Sixteen percent of PPI prescribing was generic.

Table 2 presents the potential annual net ingredient cost savings for a one year period (2007–2008) for each of the five scenarios based on prescribing guidelines [8,9]. The greatest cost savings were obtained by switching patients’ PPI therapy to the least expensive PPI and also stepping patients down to maintenance dose after 3 consecutive months at maximum therapeutic dosage (Scenario 4: 46% reduction). Costs were reduced by one third if patients were changed to an equivalent cheaper or generic brand of PPI while continuing on their original dose and quantity (Scenario 2).

**Table 2:** Estimated annual net ingredient cost (NIC) savings € and % reduction as a proportion of overall NIC for effective and economical PPI prescribing (5 scenarios)

***Scenarios (1 to 5)***	***Cost Savings (NIC)***	**% *Overall (NIC)***
**1** PPI initiation (least expensive brand)	€36,943,348	41.91
**2** Therapeutic switching (cheaper brand, same dose)	€29,568,475	33.54
**3** Dose reduction (maintenance)	€21,289,322	24.15
**4** Therapeutic switching and dose reduction	€40,505,013	45.95
**5** Therapeutic substitution (H2 Antagonist)	€34,991,569	39.69

In clinical practice it is likely that dose reduction and therapeutic substitution will only be obtained for a proportion of prescriptions. Potential costs savings for Scenario 2 were €17 million (25%) based on a PPI therapeutic substitution rate of 60% in a one year period (2007–2008) [27]. Potential cost savings for Scenario 3 ranged from €8 to €15 million (12-21%) based on 40-70% of patients successfully stepping down to the maintenance dose of their existing PPI for a one year period (2007–2008) [8,9,28].

### Potential cost savings by age group

**Figure 2:**
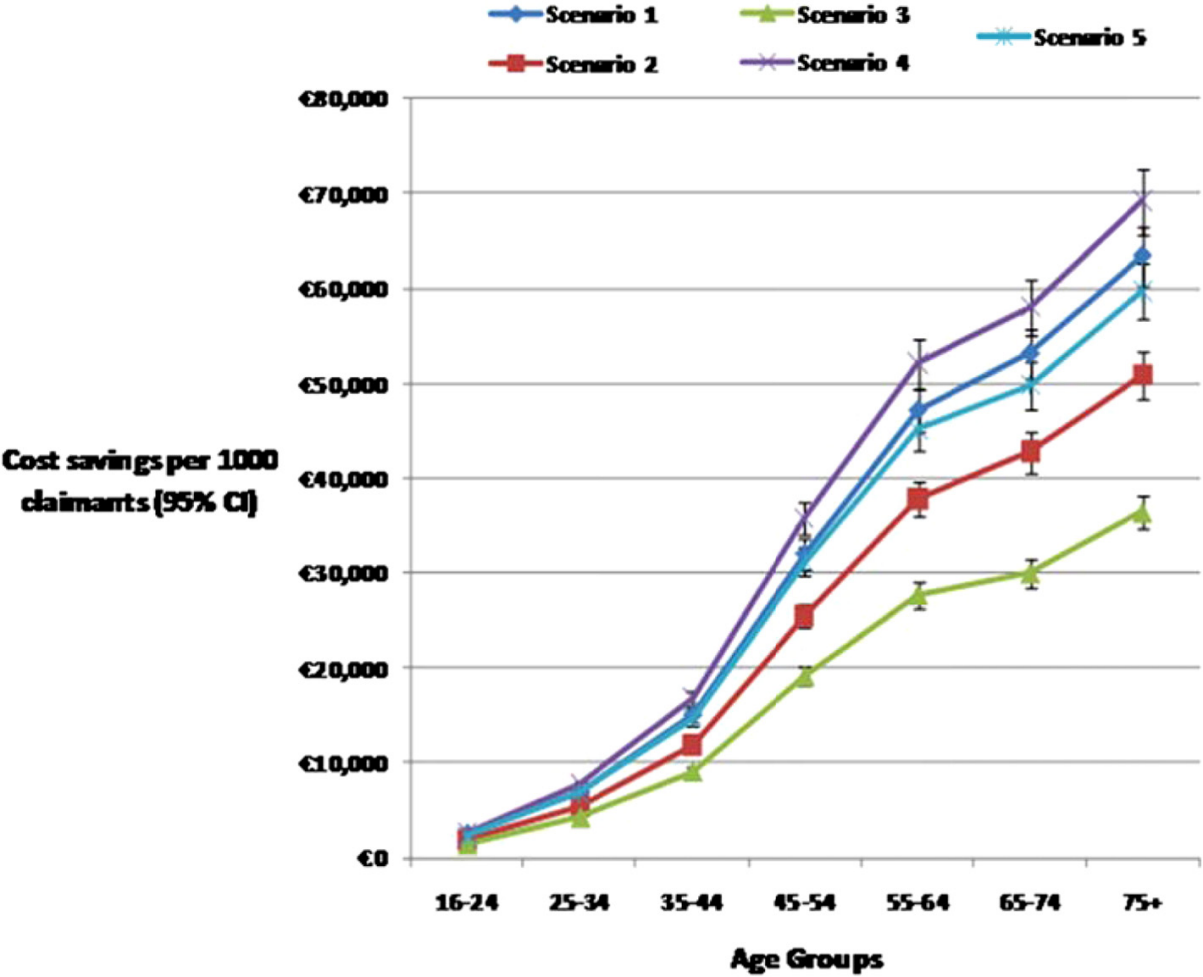
**Estimated annual net ingredient cost savings (€ per 1000 claimants) for the 5 scenarios by age distribution of the HSE-PCRS population. Notes**: Scenario 1 – Least expensive PPI at initiation. After 3 months of initial therapy: Scenario 2- Therapeutic switching (cheaper brand/generic equivalent), Scenario 3- Dose reduction (maintenance therapy), Scenario 4- Therapeutic switching and dose reduction and Scenario 5- Therapeutic substitution.

Cost savings for all five scenarios were highest in the older age groups (65 years and older). The average rate of change in cost savings increased by 90% to 105% between the 45 to 54 years and 75 years and older age group across all five scenarios (Figure 2).

## Discussion

### Principal findings

The extent to which patients continue on long term PPI treatment is a significant contributor to cumulative prescribing volume and cost and has a considerable impact on prescribing budgets. The five scenarios suggest effective ways of reducing PPI prescribing based on current guidelines and identify substantial savings in a national community drugs scheme [8,9]. The greatest cost savings were obtained by switching patients’ PPI therapy to the least expensive PPI and also stepping patients down to a maintenance dose after 3 consecutive months at maximum therapeutic dosage (Scenario 4).

The guidelines recommend regular review of patients to assess their continuing need for PPIs and the use of step-down therapy. Maintenance therapy is indicated for duodenal ulceration, non-steroidal anti-inflammatory drug (NSAID) induced ulceration and gastro oesophageal reflux disease (GORD). A regular maintenance low dose of most PPIs will prevent recurrent GORD symptoms in 70-80% of patients [8,9,29]. The analysis of the HSE-PCRS pharmacy claims database indicated that the majority of patients on long term PPI therapy were continuously prescribed maximum therapeutic dose; costs were potentially reduced by one quarter with use of step down therapy. PPI prescribing was most prevalent in the older age groups (≥65 years) where there is an increased risk of drug interactions and polypharmacy [28].

Studies have identified prescribing problems at the interface between primary and secondary care. In the UK, 40% of patients initiated on a PPI during hospital admission were not reviewed for the continuing need for PPI therapy on discharge and less than one-third of discharge letters suggested a review date to the primary care physician [15]. In New Zealand, 71% of patients discharged on PPIs without appropriate indication, continued on PPI therapy for 6 months or longer [13]. Primary care providers may be reluctant to discontinue medications prescribed by hospital specialists due to the lack of information provided by the hospital on discharge, although specialists assume that step-down therapy will be attempted; in a US Veterans primary care centre, 88% of patients had no documented attempt at step-down therapy [28]. In Norway, hospital specialists are required to verify diagnosis and recommend therapy before PPIs are reimbursed [30].

Research in health care settings indicates that patients are able to adopt step-down therapy and discontinue PPI use successfully. In the US 48% of Veterans adopted step-down therapy , while 58% of patients on long-term PPI therapy discontinued PPI use and remained asymptomatic with no significant change in quality of life after 1 year [28,31]. In Europe, 24% of Dutch and 37% of Swedish patients stopped or reduced their use of PPIs with no impact on symptom severity and quality of life [32,33]. Potential savings in Ireland for a one year period (2007–2008) were estimated based on the evidence that 40-70% of patients have successfully managed to step-down to a maintenance dose of their existing PPI in practice [8,9,28].

The guidelines also recommend prescribing the least expensive PPI or treatment [8,9]. Given the superior efficacy of PPIs compared to other acid inhibiting agents, therapeutic substitution may not be an acceptable option for many patients [4,5]. Therapeutic switching to an equivalent cheaper or generic PPI provides a method of cost control that does not affect the quality of patient care. In the UK, 64% of prescription items were dispensed generically in 2007 while in Ireland 19% were dispensed generically and 25% of prescription items were dispensed as a proprietary preparation when a generic equivalent was available [34,35]. This study has shown that increased generic prescribing of PPIs has the potential to produce significant savings. In Sweden, 60% of total possible savings were achieved during the first year of a generic substitution scheme [27] and cost savings for a similar proportion of PPI therapeutic substitution in practice in Ireland, were estimated for a one year period (2007–2008).

### Strengths and limitations

This study has a number of possible limitations. The lack of detailed diagnostic information that determines clinical indications for PPI therapy in the database limited the investigation of individual patient factors and differences in drug indication. It is likely that further savings could be achieved by discontinuing PPI therapy for some individuals where appropriate [31,32]. On demand and intermittent PPI therapy have also been shown to be cost-effective in some cases [36,37]. Notwithstanding the limitations, this study has identified significant potential cost savings based on current guidelines which could be used to provide feedback and comparative information at practice or physician level enabling changes in prescribing practices that optimise patient treatment while controlling costs [8,9].

### Future research

This study identifies potential cost savings in a national community drugs scheme but it does not account for the costs of implementing such a system of change. A review of patients on long term PPI therapy is time consuming and needs to be facilitated by prescription software systems to generate patient-specific assessments and prescribing advice and support which enable practitioners to adequately monitor dose and duration of treatment. Multicentre studies, accounting for the effect of implementing a system of PPI substitution and dose reduction on patient outcomes as well as actual cost savings over time is required. Patients’ acceptability of switching from their original branded PPI to a cheaper or generic PPI need to be considered, though previous research indicates a high proportion of patient acceptability [27].

### Policy implications

While there is evidence that patients are able to adopt step-down therapy and discontinue PPIs successfully, physicians still need to be motivated to adopt guidelines or changes in prescribing practices. Guidelines need to be closely monitored and prescribers may need to be either educated and/or persuaded to comply with them. Studies using educational interventions and academic detailing for general practitioners have had varied success. A UK study which disseminated passive written guidelines reported no effect on the proportion of patients prescribed a PPI, while a multifaceted participatory educational strategy involving workshops, guidelines and reminders reported a 60% reduction in PPI costs [10,38]. National UK initiatives such as the “Better Care, Better Value” indicator have also been successful in increasing generic PPI utilisation [39].

Financial incentives can be effective in influencing the prescriber’s choice of drug and may be incorporated into physician budgets or guidelines. An Italian study which linked implementation of dyspepsia guidelines to a pay deal for general practitioners reported a 26% reduction in PPI expenditure in comparison with non-participating practices [40]. France and Sweden, have introduced prescribing targets such as the percentage of prescriptions for generic PPIs linked with financial incentives [41,42]. Physician PPI prescribing patterns may also be benchmarked against each other with financial penalties for excessive costs [24].

Many European countries have introduced policies of generic substitution and reference pricing systems, which include incentives and regulations to encourage prescription and/or substitution of cheaper generic products for branded products [43]. In a reference pricing system, the healthcare payer will reimburse a fixed price for a group of interchangeable medicines. If a more expensive interchangeable medicine is prescribed the patient is required to pay the difference in price. The reference pricing system has recently been proposed in Ireland [43,44]. In recent years, smaller European countries, such as Lithuania, Norway and Sweden have been successful in engineering generic price reductions with drug manufacturers [45]. New drug pricing agreements have recently been implemented in Ireland between the manufacturers and the government and provide for price cuts of up to 40% on all long established, post patent medications with a generic equivalent on the market, bringing further potential savings [46].

## Conclusion

PPIs are highly effective for a wide range of acid-peptic conditions but the evidence suggests they are being over prescribed in Ireland for longer durations and at higher doses than current guidelines advise. At a time of growing concern over rising drug costs and limited health care resources potentially inappropriate or unnecessary use of expensive drugs like PPIs should be limited where possible [34]. Many patients with gastro-intestional disorders have legitimate needs for PPI to enhance their quality of life; however, this analysis highlights the potential cost savings that could be obtained with limited impact on clinical outcomes.

As PPIs lose patent protection and cheaper generic equivalents become available on the market, cost savings will increase. However, unless an incentive is introduced to promote increased generic drug utilisation and physicians are motivated and supported in changing their prescribing practices it is unlikely that the trends in PPI prescribing reported in this study will change and potential cost savings will be realised.

## Competing interests

The authors declare that they have no competing interests.

## Authors’ contributions

CC and KB and CT planned and designed the study. CC, KB, CT, LT and TF interpreted the guidelines and their application to the prescribing database. CC drafted the manuscript. KB, TF, CT and LT critically reviewed and approved the final manuscript. KB is guarantor. All authors read and approved the final manuscript.

## Authors’ information

CC: HRB PhD Scholar in Health Service Research. TF: Professor of General Practice, Royal College of Surgeons in Ireland. LT: Chief Pharmacist at the National Centre for Pharmacoeconomics. CT: Statistician at the Health Information and Quality Authority. KB: Senior Lecturer in Pharmacoepidemiology, Trinity College Dublin.

## Pre-publication history

The pre-publication history for this paper can be accessed here:

http://www.biomedcentral.com/1472-6963/12/408/prepub
